# Herbal Therapies for Type 2 Diabetes Mellitus: Chemistry, Biology, and Potential Application of Selected Plants and Compounds

**DOI:** 10.1155/2013/378657

**Published:** 2013-04-04

**Authors:** Cicero L. T. Chang, Yenshou Lin, Arlene P. Bartolome, Yi-Ching Chen, Shao-Chih Chiu, Wen-Chin Yang

**Affiliations:** ^1^Department of Veterinary Medicine, National Chung Hsing University, Taichung 402, Taiwan; ^2^Department of Life Science, National Taiwan Normal University, Taipei 116, Taiwan; ^3^Institute of Chemistry, University of the Philippines, Diliman, Quezon City 1100, Philippines; ^4^Agricultural Biotechnology Research Center, Academia Sinica, No. 128, Academia Sinica Road, Section 2, Nankang, Taipei 115, Taiwan; ^5^Graduate Institute of Immunology, China Medical University, Taichung 404, Taiwan; ^6^Center for Neuropsychiatry, China Medical University Hospital, Taichung 404, Taiwan; ^7^Institute of Pharmacology, Yang-Ming University, Taipei 112, Taiwan; ^8^Institute of Zoology, National Taiwan University, Taipei 106, Taiwan; ^9^Department of Life Sciences, National Chung Hsing University, Taichung 402, Taiwan

## Abstract

Diabetes mellitus has been recognized since antiquity. It currently affects as many as 285 million people worldwide and results in heavy personal and national economic burdens. Considerable progress has been made in orthodox antidiabetic drugs. However, new remedies are still in great demand because of the limited efficacy and undesirable side effects of current orthodox drugs. Nature is an extraordinary source of antidiabetic medicines. To date, more than 1200 flowering plants have been claimed to have antidiabetic properties. Among them, one-third have been scientifically studied and documented in around 460 publications. In this review, we select and discuss blood glucose-lowering medicinal herbs that have the ability to modulate one or more of the pathways that regulate insulin resistance, *β*-cell function, GLP-1 homeostasis, and glucose (re)absorption. Emphasis is placed on phytochemistry, anti-diabetic bioactivities, and likely mechanism(s). Recent progress in the understanding of the biological actions, mechanisms, and therapeutic potential of compounds and extracts of plant origin in type 2 diabetes is summarized. This review provides a source of up-to-date information for further basic and clinical research into herbal therapy for type 2 diabetes. Emerging views on therapeutic strategies for type 2 diabetes are also discussed.

## 1. Impact and Pathogenesis of Type 2 Diabetes 

Diabetes mellitus is a chronic metabolic disease with life-threatening complications. The International Diabetes Federation (IDF) estimates that 285 million people, 6.4% of the world population, suffered from diabetes in 2010 and this prevalence will increase to 439 million people, 7.7% of the world population by 2030 [1]. Over 90% percent of diabetic patients are diagnosed with type 2 diabetes (T2D) [2, 3]. The cost of health care associated with diabetes continues to grow and is a huge economic burden for afflicted patients and countries. In 2007, approximately 17.5 million adults were reported to be receiving treatment for diabetes in the USA, where the estimated cost of diabetes was US 174 billion dollars [4]. 

**Figure 1:**
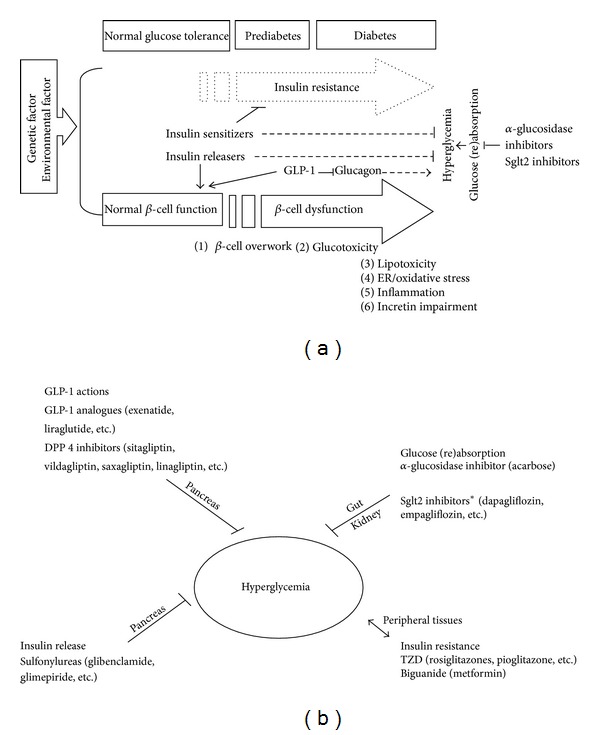
Etiology, development, and current therapies for T2D. (a) Genetic and environmental factors are the main contributors to the development of insulin resistance and impaired glucose tolerance. Under normal glucose tolerance conditions, *β* cells secrete insulin in response to a surge in glucose after a meal. At the initial stage, *β* cells overwork to compensate for the development of insulin resistance. Later on, *β* cells are no longer able to secrete enough insulin to overcome insulin resistance. As a result, glucose tolerance is impaired and the disease progresses from prediabetes to diabetes. Diabetes is characterized as a loss of blood glucose homeostasis, a condition termed hyperglycemia, in the patients. Glucotoxicity, lipotoxicity, ER/oxidative stress, inflammation, and incretin impairment are risk factors for *β*-cell dysfunction. Besides insulin, insulin releasers, insulin sensitizers, GLP-1 analogues/DDP-4 inhibitors, and a-glucosidase inhibitors and Sglt 2 inhibitors are common antidiabetic drugs. (b) Insulin releasers (e.g., sulfonylureas such as glibenclamide and glimepiride) can stimulate pancreatic *β* cells to secrete insulin. Insulin sensitizers (TZDs (e.g., rosiglitazone and pioglitazone) and biguanide (metformin)) reduce insulin resistance in the peripheral tissues. GLP-1 has multiple direct actions on pancreas (insulin and glucagon production) and gastric emptying. Injection of exogenous GLP-1 (e.g., exenatide and liraglutide) or inhibition of endogenous GLP-1 degradation by DPP-4 inhibitors (e.g., sitagliptin, vildagliptin, saxagliptin, and linagliptin) can maintain GLP-1 levels. Inhibitors of *α*-glucosidases (acarbose) and Sglt 2 (e.g., dapagliflozin and empagliflozin) reduce glucose absorption in guts and glucose reabsorption in kidney, respectively. All the drugs can diminish hyperglycemia. *Sglt 2 inhibitors were disproved by the FDA because of a safety issue.

Despite considerable progress in scientific studies on T2D and research and development of antidiabetic agents, the cause of T2D is not yet fully understood. Mounting evidence from epidemiological studies suggests that genetic and environmental factors are primary causes of T2D. Both factors contribute to insulin resistance and loss of *β*-cell function that result in impairment in insulin action, insulin production, or both. This impairment results in the development of hyperglycemia, a major pathological feature of T2D [5]. Such hyperglycemia is detrimental to *β* cells and peripheral tissues, a condition termed glucotoxicity, which is clinically relevant as a cause of diabetes-related complications such as cardiovascular disease, nephropathy, retinal blindness, neuropathy, and peripheral gangrene [6]. Therefore, maintenance of glycemic homeostasis is the most common therapeutic aim for patients with T2D. Moreover, aberrant lipid metabolism in adipose and other tissues can cause lipotoxicity, which can further worsen diabetic complications. The *β* cells in the pancreas are the key players in glycemic homeostasis. Glucotoxicity, lipotoxicity, endoplasmic reticulum (ER)/oxidative stress, inflammatory mediators, and incretin were reported to modulate *β*-cell function and survival [7]. The relationship between the causes and pathogenesis of T2D is illustrated in Figure 1(a). 

Insulin is a protein hormone that regulates the metabolism of glucose, fat, and protein in the body [8]. Any defect in insulin production and action leads to serious metabolic problems. Pancreatic *β* cells are the only endocrine cells known to produce insulin. In the development of T2D, *β*-cell function in the presence of increasing hyperglycemia and insulin resistance declines. This decline may begin early in the disease and accelerates after compensatory overwork that drives the progression of T2D. Targeting *β*-cell failure early in disease progression has evolved as a new approach to treat T2D [9]. Currently, no antidiabetic drugs have been proven clinically effective for the prevention of *β*-cell atrophy although thiazolidinediones (TZDs) and glucagon-like peptide-1 (GLP-1) analogues have reported to be effective in animals [10–12]. Therefore, maintenance and enhancement of *β*-cell function have the potential to stabilize, delay, and even reverse T2D [7]. Specific growth factors, cell cycle mediators, and nuclear factors have been proposed to regulate *β*-cell homeostasis [13, 14]. New therapeutic classes of diabetes medications that act to regulate *β*-cell function could be clinically potent in reversing the disease.

Insulin resistance is a condition whereby the body's cells become resistant to the action of insulin. Insulin resistance usually emerges many years before the onset of T2D as a result of interplay between genetic and environmental factors [15]. Metabolic hormones (e.g., leptin, adiponectin, and glucagon), nutrient excess, systemic free fatty acids, ER stress/oxidative stress, adipose hypoxia, adipose inflammation, and so on account for the generation of insulin resistance [16]. At the molecular level, fatty acid derivatives, inflammatory mediators, and free radicals trigger a negative regulation of IRS-1, PI3K, Akt, GSK-3*β*, JNK, and other mediators downstream of insulin receptors.

Loss of glycemic control in T2D patients is an undesirable and detrimental consequence. Therefore, normalization of blood glucose by reducing glucose absorption from the gut and kidney is effective to control glycemic homeostasis in diabetic patients. Reduction of dietary saccharides or inhibition of glucose from oligosaccharide degradation by *α*-glucosidases in intestines helps to diminish blood glucose level. Contrarily, the renal tubule can recover 90% of urine glucose, contributing to glucose homeostasis in the body. Since sodium glucose cotransporter 2 (Sglt 2) is primarily expressed in kidney tubules, an Sglt 2 inhibitor was developed as antihyperglycemic agent. *α*-Glucosidase inhibitors and Sglt 2 inhibitors have been demonstrated to be clinically effective against hyperglycemia. Nonetheless, Sglt 2 inhibitor was not approved by the US Food and Drug Administration (FDA) because of safety concerns [17].

Incretin consists of glucose-dependent insulinotropic polypeptide (GIP) and glucagon-like peptide-1 (GLP-1), which are generated by enteroendocrine K-cells and L-cells, respectively [18, 19]. GLP-1 plays a dominant role in modulating *β*-cell function (insulin production and *β*-cell proliferation/protection), reducing glucagon secretion, attenuating gastric emptying, and decreasing appetite/weight gain [20]. Accordingly, the action of incretin is impaired in T2D [21–23]. GLP-1 has a short half life (<2 min) due to its fast cleavage by dipeptidyl peptidase-4 (DPP-4) [24, 25]. Therefore, GLP-1 mimetics and DPP-4 inhibitors have been approved by the FDA as new classes of antidiabetic drugs [26, 27]. 

More information on the molecular mechanisms underlying the pathogenesis of T2D is required for further clinical success.

## 2. Pharmacotherapies for T2D and the Related Challenges 

T2D arises from a defect in insulin secretion, insulin action, and/or both. Hence, T2D therapy has evolved from monotherapy using insulin, insulin secretagogues, or sensitizers alone to combination therapy using insulin/insulin secretagogues plus sensitizers and/or incretin-based drugs. The mechanisms of action implicated in these remedies include insulin production, sensitization of the insulin receptor pathway, and/or GLP-1 secretion. The more pathways the drugs target, the better the clinical outcome and therapy seem to be. More recently, manipulation of *β* cells *per se* or in combination with other antidiabetic therapeutics has emerged as a new strategy to ameliorate and, particularly, cure T2D [7].

By far the most popular approach to treating T2D is glycemic control in an attempt to reduce complications and death. When pharmacological methods are used to interfere with these mechanisms, the percentage of glycosylated hemoglobulin A_1c_ (HbA_1c_), an indicator of long-term glycemic control, in medicated T2D patients is suggested to be below 7%. Since hyperglycemia is implicated in diabetic complications and death in patients, better regulation of glycemic maintenance ameliorates progression and severity of T2D. In the past, several drugs for T2D including oral antidiabetic agents (OAAs), insulin, and incretin-based drugs have been developed to control homeostasis of blood sugar via different mechanisms (Figures 1(a) and 1(b)) [28]. Among OAAs, insulin releasers such as sulfonylurea-type drugs (e.g., glibenclamide and glimepiride) can directly stimulate pancreatic *β* cells to secrete insulin, leading to lower blood glucose. However, these secretagogues cannot rescue *β*-cell atrophy. In contrast, insulin sensitizers such as TZDs (e.g., rosiglitazone and pioglitazone) and a biguanide, metformin, can directly lower insulin resistance and, subsequently, blood glucose. Glucose (re)absorption is viewed as an alternative way to lower blood glucose level. Inhibitors of Sglt 2, dapagliflozin, empagliflozin, and *α*-glucosidase inhibitor, acarbose, inhibit the activity of sodium-glucose cotransporter-2 and *α*-glucosidases, respectively, leading to a decrease in glucose (re)absorption via the renal tubules and the intestine, respectively [17, 29]. Another new class of diabetes therapeutics is the GLP-1 analogues (exenatide and liraglutide) and DPP-4 inhibitors (e.g., sitagliptin, vildagliptin, saxagliptin, and linagliptin). This class leverages multiple actions of GLP-1 to lower blood glucose, including increasing insulin, reducing glucagon, and gastric emptying [30]. Notably, insulin is still an effective drug for T2D. Aside from medication, the importance of diet and lifestyle management in preventing and alleviating T2D should not be neglected.

But current antidiabetic agents lack efficacy and also have undesirable side effects [31]. For instance, insulin secretagogues are frequently linked with weight gain, hypoglycemia, and inability to protect *β* cells from death [28, 32]. TZDs and biguanides result in weight gain and kidney toxicity, respectively. Acarbose usually causes gastrointestinal upset such as diarrhea and flatulence. Additionally, a clinical trial of Sglt 2 recently failed due to safety concerns [17]. Despite the multiple benefits of incretin-based drugs, these drugs are still accompanied by severe gastrointestinal problems such as sour stomach, belching, nausea, vomiting, indigestion, and diarrhea [29]. Even in well-managed patients, daily injection of insulin cannot match the natural precise timing and dosing of insulin secretion from the pancreas in response to hyperglycemia, resulting in severe complications. 

Moreover, in addition to lack of efficacy and undesirable adverse effects, all current antidiabetic agents have a major shortcoming in that they are only designed to alleviate T2D and not to cure it. Evidence suggests that targeting one or two metabolic pathways is insufficient to cure T2D. Drugs with the ability to target more metabolic pathways seem to show more encouraging results than those that target a single pathway, as evidenced by GLP-1. But it should also be noted that drugs that are effective in targeting metabolic pathway(s) are often associated with adverse effects [33]. To ensure patients' welfare, there is still an obvious need to develop antidiabetic medicines with satisfactory efficacy and no severe adverse effects. 

## 3. Herbal Therapy for T2D

Long before the birth of orthodox Western medicine, medicinal herbs were applied to treat a wide range of disease categories [34]. Due to emphasis on scientism and other complicated reasons, Western medicine now prevails over “traditional” forms of medicine including herbal medicine systems. Although herbal medicine systems are sometimes misinterpreted as being unscientific and anachronistic, their long-term existence proves they are able to compete with Western drugs at some level. The use of a medicinal herb, alone or in combination with other herbs, can be thought of as a type of combination therapy because of the complexity of the phytochemicals and bioactivities in the plant. Thus, a single antidiabetic herb with thousands of phytochemicals may have multiple benefits by targeting several metabolic pathways and essentially “killing several birds with one stone.” One study supported this principle by demonstrating that a combination therapy of orthodox medicine and herbal medicine exhibited a better (synergistic) effect than either medicine alone [35]. Therefore, herbal medicine can complement orthodox therapy in T2D and provides hope for a cure. 

**Figure 2:**
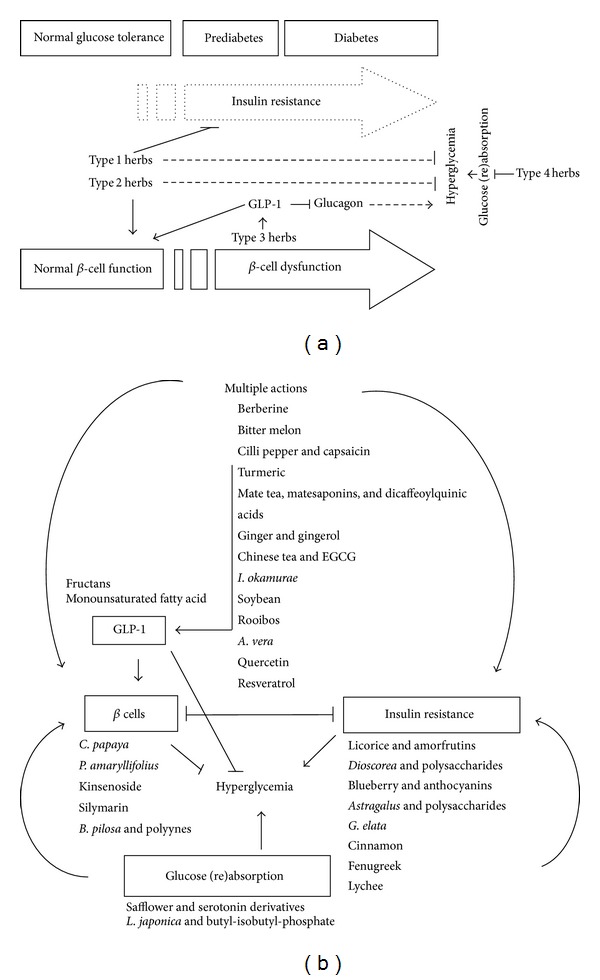
Mechanisms underlying herbal therapies using antidiabetic plants and phytocompounds. (a) Different types of medicinal herbs can be classified based on their modes of action such as insulin resistance (type 1 herbs), *β*-cell function (type 2 herbs), and GLP-1 (type 3 herbs) and glucose (re)absorption (type 4 herbs). (b) The selected plants and compounds exert their antihyperglycemic effect through targeting one single mechanism (insulin resistance (type 1 herbs), *β*-cell function (type 2 herbs), GLP-1 (type 3 herbs), or glucose (re)absorption (type 4 herbs)) or multiple mechanisms.

Medicinal herbs have never become obsolete and still play a prominent role in human health care. Among them, over 1200 plants have been claimed to be remedies for diabetes [36, 37]. Over 400 plants as well as 700 recipes and compounds have been scientifically evaluated for T2D treatment [38]. Metformin was developed based on a biguanide compound from the antidiabetic herb, French lilac, and is now a first-line drug for T2D [39]. Medicinal herbs contain diverse bioactive compounds and can have multiple actions on insulin action, insulin production, or both. In the present review, we focus on scientific studies of selected glucose-lowering herbs and phytocompounds and their ability to target insulin resistance, *β*-cell function, incretin-related pathways, and glucose (re)absorption (Figures 2(a) and 2(b)). Phytochemistry, antidiabetic bioactivities, and likely modes of action of the selected plants and compounds are discussed.

## 4. Selected Medicinal Herbs and Compounds for T2D

More than 400 plants and compounds have shown antidiabetic activities *in vitro* and/or *in vivo*. Instead of listing each extract/compound, here, we select some plant chemicals and/or extracts with the ability to control blood glucose as well as to modulate at least one of the following mechanisms involved in insulin resistance: *β*-cell function, incretin-related pathways, and glucose (re)absorption. Chemical structure, antidiabetic activity and action in cells, animal models, and the results of administration of the plant extracts and compounds to patients of T2D are discussed. The chemical and biological properties of the compounds discussed in this section are summarized in Table 1.

### 4.1. Herbs and Compounds That Regulate Insulin Resistance

#### 4.1.1. Amorfrutins and Licorice

Licorice, the common name for the plants that comprise the genus *Glycyrrhiza*, is utilized as herbal medicine for a wide range of diseases. The ethanol extract of *G. uralensis* was found to reduce blood glucose, fat weight, and blood pressure in rodent models [128]. This extract also has PPAR-*γ* activity [128]. Further, amorfrutins isolated from the licorice, *G. foetida*, were found to bind to and activate peroxide proliferator-activated receptor-*γ* (PPAR-*γ*), a central player in glucose and lipid metabolism [40]. These compounds lowered blood glucose, fat weight, and dyslipidemia [40] indicating that licorice and its active amorfrutins exert their antidiabetic function via the PPAR-*γ* pathway.

#### 4.1.2. *Dioscorea* Polysaccharides and *Dioscorea*

The rhizome of *Dioscorea* is used as a traditional Chinese medicine for asthma, abscesses, chronic diarrhea, and ulcers [41]. Several studies on rodent models of diabetes have reported that *Dioscorea* extract improves glycemic control and insulin resistance [41–44]. Further, *Dioscorea* extract reduced blood glucose in high fat diet-induced rats [41]. The antidiabetic mechanism of *Dioscorea* extract involves reduction of insulin resistance by diminution of the phosphorylation of ERK and pS6K and increase of the phosphorylation of Akt and glucose transporter 4 (Glut4) [41]. Another study demonstrated that *Dioscorea* polysaccharides reduced insulin resistance mediated by inflammatory cytokines as evidenced by the phosphorylation of insulin receptor substrate (IRS) and Akt [42].

#### 4.1.3. Anthocyanins and Blueberry

Blueberry (*Vaccinium* spp.) was demonstrated to lower systolic and diastolic blood pressure and lipid oxidation and improve insulin resistance, diabetes, diabetic complications, and digestion [45, 129–132]. Notably, blueberries contain powerful antioxidants that can neutralize free radicals that cause neurodegenerative disease, cardiovascular disease, and cancer [45]. Accordingly, phenolics and anthocyanins were proposed as active compounds for diabetes and insulin resistance [45, 46].

One clinical study showed that obese or T2D patients consuming 22.5 g blueberry, twice a day for 6 weeks, reduced insulin resistance to a greater extent than those consuming a placebo [47]. The data confirm the beneficial effect of blueberry on metabolic syndrome. However, the active compounds related to this claim need further investigation.

#### 4.1.4. *Astragalus* Polysaccharides and *Astragalus*

The root of *Astragalus membranaceus* has long been used as a Chinese medicine and shows antioxidant, antidiabetic, antihypertensive, and immunomodulatory activities [133]. The extract of *A. membranaceus* was shown to treat diabetes and diabetic complications [134]. Moreover, treatment with *Astragalus* polysaccharides resulted in better glycemic control in diabetic rodents via an increase in insulin sensitivity [48–50]. The mode of action of *Astragalus* polysaccharides includes Akt activation and upregulation of Glut4 and inhibition of inflammation via the PTP1B/NF*κ*B pathway [48, 50, 51].

#### 4.1.5. *Gastrodia elata*

*G. elata* has been utilized as Chinese medicine for blood circulation and memory [52]. More recently, the extract of *G. elata* has been reported to improve insulin resistance [52]. Vanillin and 4-hydroxybenzaldehyde were proposed as the active compounds. Both compounds reduced insulin resistance through a decrease in fat accumulation in adipose tissues and an increase in fat oxidation and potentiation of leptin signaling in obese rats [52]. So far, no clinical study has been conducted in human diabetic patients.

#### 4.1.6. Cinnamon

Both common cinnamon (*Cinnamomum verum* and *C. zeylanicum*) and cassia (*C. aromaticum*) have long been used as flavoring agents and in drinks and medicines worldwide [135]. Cinnamon has traditionally been used for rheumatism, wounds, diarrhea, headaches, and colds [136]. Recently, extensive studies have been performed on the action of cinnamon on diabetes and metabolic syndrome [135]. Cinnamon was shown to reduce blood glucose via reduction of insulin resistance and increase of hepatic glycogenesis [135, 137]. Cinnamon phenolics were proposed to be the active compounds in modulation of insulin signaling [53, 138, 139]. Moreover, cinnamaldehyde had antihyperglycemic and antihyperlipidemic effects on rodent models of diabetes [53]. This compound from cinnamon extract is thought of as a potential antidiabetic agent [139]. Unfortunately, the molecular target of cinnamon and cinnamaldehyde remains unclear.

#### 4.1.7. Fenugreek

The seeds of fenugreek (*Trigonella foenum-graecum*) are used as a food supplement and also have a long history of medicinal use for labor induction, helping digestion and improving metabolism and health [34]. Animal studies have shown that extract of fenugreek seeds can lower blood glucose levels [140, 141]. Fenugreek is considered a promising agent for diabetes and its complications [34]. The glucose-lowering action of this plant involves reduction of insulin resistance [142]. Diosgenin, GII, galactomannan, trigoneosides, and 4-hydroxyisoleucine have been identified as the active antidiabetic compounds in fenugreek. However, little is known about the mechanisms of these compounds [55]. Among them, diosgenin was shown to reduce adipocyte differentiation and inflammation, implying its action in reduction of insulin resistance [54]. A clinical study indicated that fenugreek exerts hypoglycemic control via increasing insulin sensitivity [143].

#### 4.1.8. Lychee

Lychee (*Litchi chinensis*) is an evergreen fruit tree. Its seeds are used as a Chinese herbal medicine for pain, gastrointestinal diseases, and others. Recently, lychee seed was reported to have antidiabetic activity in rats [56] and human patients [144]. Lychee seed extract exerts its action through reduction of insulin resistance [56]. In addition, oligonol from lychee fruit showed anti-oxidative activity and, thus, protected the liver and kidney in T2D animal models [57, 58].

### 4.2. Herbs and Compounds That Regulate *β*-Cell Function

In this section, plant chemicals and/or extracts are listed according to their impact on *β* cells. Their chemical structures and antihyperglycemic activities and actions on *β*-cell function ((re)generation and survival) in cells, animals, and T2D patients are discussed. The chemical and biological properties of the compounds discussed in this section are summarized in Table 1.

#### 4.2.1. *Carica papaya* and *Pandanus amaryllifolius*

The ethanol extracts of *P. amaryllifolius* and *C. papaya* reduced hyperglycemia in streptozotocin- (STZ-) treated mice [59]. Histological staining data showed that these extracts significantly induced the regeneration of the *β* cells as evidenced by reduced blood glucose level [59]. So far, no active components have been identified. However, the flavonoids, alkaloids, saponin, and tannin in both plants were speculated to be bioactive phytochemicals [59].

#### 4.2.2. Conophylline and *Tabernaemontana divaricata*

Conophylline, a plant alkaloid present in *T. divaricata* or *Ervatamia microphylla*, facilitates differentiation and generation of pancreatic *β* cells *in vitro* and *in vivo* [60–62]. This phytochemical was also shown to decrease the fibrosis of pancreatic islet cells [63]. Crude extract of *T. divaricata* was able to increase the level of blood insulin and reduce the level of blood glucose in STZ-treated mice [145]. These data imply a plausible role for conophylline and *T. divaricata* in *β*-cell function.

#### 4.2.3. Kinsenoside

Kinsenoside, a major constituent of *Anoectochilus roxburghii*, exhibited hypoglycemic activity in STZ-treated mice [64]. This effect was partially attributed to *β*-cell repair and/or regeneration. However, the clinical potential of this compound in *β*-cell survival and regeneration awaits further investigation. 

#### 4.2.4. Nymphayol

Nymphayol, a plant sterol, was initially isolated and identified from *Nymphaea stellata*. One study showed that this compound promoted the partial generation of pancreatic islet cells [65]. Oral administration of Nymphayol significantly diminished the blood glucose level and increased the insulin content in diabetic rats. In addition, Nymphayol increased number of *β* cells enormously [65]. However, the impact of this compound on T2D patients is largely unknown. 

#### 4.2.5. Silymarin

Silymarin is a flavonoid mixture composed of silybin, silydianin, and silychristin, which are active components of the plant milk thistle (*Silybum marianum*) [146]. Aside from antioxidant, anti-inflammatory, and hepatoprotective activities, the modes of action through which silymarin and/or milk thistle exert antidiabetic activity are not well understood [66–74]. It has been reported that silymarin can rescue *β*-cell function in alloxan-treated rats [68].

#### 4.2.6. Polyynes and *Bidens pilosa*

*B. pilosa* is used as an herbal medicine for a variety of diseases. Ubillas and colleagues showed that the aqueous ethanol extract of the aerial part of *B. pilosa* lowered blood glucose in db/db mice [75]. Based on a bioactivity-guided identification, 2 polyynes, 3-ß-d-glucopyranosyl-1-hydroxy-6(*E*)-tetradecene-8,10,12-triyne, and 2-ß-d-glucopyranosyloxy-1-hydroxy-5(*E*)-tridecene-7,9,11-triyne were identified. Further, a mixture of both compounds significantly reduced blood glucose levels and food intake in db/db mice [75]. Another study confirmed that water extracts of *B. pilosa* at one and multiple doses significantly lowered fasting and postmeal hyperglycemia in db/db mice [76]. The anti-hyperglycemic effect of *B. pilosa* was inversely correlated with an increase in serum insulin levels, suggesting that BPWE acts to lower blood glucose via increased insulin production. Moreover, *B. pilosa* protected against islet atrophy in mouse pancreata. Despite the variation in the percentage of polyynes, *B. pilosa* varieties, *B. pilosa* L. var. *radiate* (BPR), *B. pilosa* L. var. *pilosa* (BPP), and *B. pilosa* L. var. *minor* (BPM) all displayed antidiabetic activity in db/db mice [76]. Another polyyne isolated from *B. pilosa*, 2-ß-d-glucopyranosyloxy-1-hydroxytrideca-5,7,9,11-tetrayne (cytopiloyne) showed better glycemic control than the previously-mentioned polyynes [53]. Mechanistic study demonstrated that similar to *B. pilosa*, cytopiloyne exerts antidiabetic function through regulation of *β*-cell function involving the increase insulin expression/secretion and islet protection [53]. Furthermore, cytopiloyne regulated *β*-cell function through a signaling cascade of calcium influx, diacylglycerol, and protein kinase C*α*. Collectively, *B. pilosa* and cytopiloyne derivatives can treat T2D via acting on *β* cells.

Like all antidiabetic drugs, cytopiloyne failed to prevent and cure diabetes completely but reduced diabetic complications [53]. Together the data also imply that combination therapy that targets multiple pathways involved in metabolism could be a better remedy for T2D. 

#### 4.2.7. *Gymnema sylvestre*

*G. sylvestre* is an Indian medicinal herb that has been used to treat diabetes for centuries. The extract of *G. sylvestre* has been shown to reduce blood glucose. Its action involves insulin secretion and (re)generation of pancreatic *β* cells in rodents [77, 78]. *G. sylvestre* increased plasma insulin and C-peptide levels and decreased blood glucose concentrations in T2D patients [79]. Collectively, this plant exerts its antidiabetic effect via regulation of *β*-cell function.

### 4.3. Herbs and Compounds That Regulate GLP-1 Homeostasis

The chemical and biological properties of plants and phytochemicals regulating GLP-1 secretion and/or DPP-4 activity discussed in this section are summarized in Table 1.

#### 4.3.1. Fructans

 The American Diabetes Association (ADA) established a link between high intake of soluble dietary fiber and improved hyperglycemia and insulin secretion in T2D patients [147]. Inulins (Raftilose) are soluble dietary fibers made of short-chain fructans present in the roots of chicory, *Agave tequilana*, *Dasylirion* spp., and so on. One study showed that inulin-type fructans could prevent obesity, steatosis, and hyperglycemia. Moreover, fructans were demonstrated to stimulate incretin secretion in the colon of rats through their fermentation [80, 81]. In addition, 5-week feeding with inulin significantly lowered body weight gain, food intake, and blood glucose levels in C57BL/6J mice [82]. An elevation of GLP-1 levels was observed in the portal vein and proximal colon [82]. It remains unclear whether fructans can enhance incretin production in humans with T2D.

#### 4.3.2. Monounsaturated Fatty Acid

Epidemiological investigations have established an association between dietary fat and T2D. A sedentary lifestyle with a diet overly high in fat usually accompanies obesity and T2D [148, 149]. However, fat was found to stimulate incretin secretion [150]. Decrease in gastric emptying, level of postprandial blood glucose and insulin, and an increase in plasma GLP-1 level were caused by ingesting fat before a carbohydrate meal in T2D patients [86]. In addition, T2D patients took control meals and control meals supplemented with olive oil (74% monounsaturated fatty acid) or butter (72% saturated fatty acid). In contrast to the control diet, both fat-rich meals induced a 5- to 6-fold increase in plasma GLP-1 and 3- to 4-fold increase in GIP [85]. However, no significant differences in the level of blood glucose, insulin, or fatty acids were observed [85]. In normal and lean Zucker rats, olive oil enhanced GLP-1 secretion, leading to improved glycemic tolerance [83, 84]. Data from humans and rodents suggest that fat, particularly unsaturated fatty acid, can stimulate GLP-1 secretion. 

### 4.4. Herbs and Compounds That Regulate Glucose Absorption in the Gut

The chemical and biological properties of plants and phytochemicals regulating *α*-glucosidase activity discussed in this section are summarized in Table 1.

#### 4.4.1. Serotonin Derivatives and Safflower

 Safflower (*Carthamus tinctorius*) seeds are used as a herbal medicine for menstrual pain, trauma, constipation, and diaphoresis in Korea and Asian countries [87]. Hydroalcoholic extract of safflower exhibited antidiabetic properties through enhancing insulin secretion in alloxan-induced diabetic rats [151]. Two serotonin derivatives isolated from safflower seed were shown to suppress *α*-glucosidase activity to a greater degree than the positive control acarbose [87].

#### 4.4.2. Butyl-isobutyl-phthalate and *Laminaria japonica*

 Rhizoid of Japanese kelp, *L. japonica*, has been used to treat diabetes. Butyl-isobutyl-phthalate, an active compound of *L. japonica*, exhibited inhibition of *α*-glucosidase activity *in vitro* and a hypoglycemic effect on diabetic mice induced by STZ [88]. 

### 4.5. Herbs and Compounds with Multiple Antidiabetic Actions

Some plants and plant compounds can target multiple metabolic pathways. The chemical and biological properties of the compounds discussed in this section are summarized in Table 1.

#### 4.5.1. Berberine

Berberine, an isoquinoline alkaloid, was first isolated from *Berberis vulgaris*. This compound has multiple functions ranging from inflammation inhibition and cancer suppression to reduction of metabolic syndrome and other activities [93, 152–155]. With respect to T2D, this compound lowered hyperglycemia, increased insulin resistance, stimulated pancreatic *β*-cell regeneration, and decreased lipid peroxidation in a mouse model of T2D [89–92]. Thus, it may be useful for treatment of T2D and other types of diabetes. A meta-analysis study suggests that berberine *per se* does not show glycemic control in T2D patients. Combination treatment of berberine with other OAAs showed better glycemic control than either treatment alone. Of note, berberine had a mild antidyslipidemic effect on patients [94].

#### 4.5.2. Bitter Melon

Bitter melon, the fruit of the plant *Momordica charantia* is used in Ayurvedic medicine [156]. The biochemistry and bioactivities associated with the antidiabetic effect of the extracts of bitter melon and *M. charantia* as a whole have been extensively studied. One *in vitro* study showed that bitter melon could increase insulin secretion from *β* cells. Moreover, immunostaining data indicated that the juice of the bitter melon increased *β* cells in the pancreas of STZ-treated rats. Modes of action of bitter melon and *M. charantia* include insulin secretion, inhibition of glucose reabsorption in guts, preservation of islet *β* cells and their functions, increase of peripheral glucose utilization, and suppression of gluconeogenic enzymes [38]. Of note, momorcharin and momordicin, isolated from *M. charantia* and its fruit, act to lower blood glucose likely because they possess insulin-like chemical structures [38].

#### 4.5.3. Capsaicin and Chili Pepper

Chili peppers, the fruits of the *Capsicum* plants, are commonly used as food and medicine. Chili pepper extract exerts an insulinotropic action, implying its action on *β* cells [157]. Capsaicin, a pungent component of chili pepper, activates AMPK in 3T3-L1 preadipocytes [95]. The data suggest that the chili pepper and its active ingredients prevent T2D via regulation of insulin resistance and probably *β* cells. However, there is a discrepancy over the use of capsaicin to treat T2D. Capsaicin might cause T2D via impairment of insulin secretion [96]. Therefore, precaution should be taken in the use of capsaicin for T2D.

#### 4.5.4. Ginseng

Ginseng (*Panax ginseng*) has been viewed as a panacea in oriental medicine. *P. ginseng* and North American ginseng (*P. quinquefolius*) were demonstrated to lower blood glucose in rodent models [158, 159]. Roots, berries, and/or leaves were found effective against T2D in humans and/or rodents [3, 160–164]. Some clinical studies have demonstrated that *P. ginseng* and North American ginseng improve glycemic control in T2D patients [165, 166]. However, another study reported that neither ginseng had an antidiabetic effect on diabetic patients [167]. This discrepancy may be the result of a variation in active ginsenosides in ginseng [168]. The glucose-lowering mechanisms of both ginsengs may involve a reduction in insulin resistance and *β*-cell function [97, 169–173]. Ginsenosides are the primary constituents present in ginseng roots that are claimed to benefit health. Extracts of ginseng root have been shown to protect against apoptosis of the pancreatic *β*-cell line, Min-6 cells [171]. One study proposed that ginseng alters mitochondrial function as well as apoptosis cascades to ensure cell viability in pancreatic islet cells [174]. Moreover, ginsenosides from ginseng extracts were reportedly responsible for this protection *in vitro*. One study reported that ginsenoside Rh2 is an active compound that improves insulin resistance in fructose-rich chow-fed rats [97]. Besides, ginsenoside Re was showed to possess antioxidant activity via upregulation of glutathione and malondialdehyde in kidney and/or eye [98]. However, the *in vivo* protective role of the extracts and ginsenosides in *β* cells remains to be further verified. 

#### 4.5.5. Turmeric

Like many spices such as garlic and ginger, turmeric shows hypoglycemic and hypolipidemic effects on diabetic mice [175]. Turmeric also increased postprandial serum insulin levels to maintain blood glucose levels in healthy subjects [99]. Curcumin is a major constituent of the rhizomatous powder of turmeric (*Curcuma longa*) and is commonly used as food and medicine in southern Asia. Curcumin and turmeric rhizomes show a number of bioactivities such as antioxidant, anti-inflammatory, antidiabetic, and immunomodulatory [176]. Curcumin has been used to treat T2D [177, 178]. Weisberg and colleagues pointed out that curcumin reverses many of the inflammatory and metabolic derangements associated with obesity and improves glycemic control in mouse models of type 2 diabetes [178]. Chuengsamarn and colleagues showed that after 9 months of treatment, a curcumin-treated group showed a better *β*-cell function, with higher homeostatic measurement assessment (HOMA)-*β* and lower C-peptide. Also, the curcumin-treated group showed a lower level of HOMA insulin resistance (IR). This study demonstrated that curcumin can prevent T2D in humans [177]. Consistently, another clinical study exhibited that the ingestion of turmeric increased postprandial serum insulin levels in healthy subjects. These data suggest that curcumin, a bioactive compound of turmeric, ameliorates T2D via regulation of insulin resistance and *β*-cell function [99]. Further, turmerin, an anti-oxidant protein identified from turmeric, was also shown to inhibit *α*-glucosidase activity [100]. Overall, turmeric exerts antidiabetic actions likely via regulation of insulin resistance, *β*-cell function, and gut absorption.

#### 4.5.6. Dicaffeoylquinic Acids, Matesaponins, and Mate Tea

Mate tea is made from the leaves of mate, *Ilex paraguariensis* (Aquifoliaceae), in South America [101]. Mate has been claimed to have neuroprotective, antioxidant, hepatoprotective, choleretic, diuretic, hypocholesterolemic, antirheumatic, antithrombotic, anti-inflammatory, antiobese, and cardioprotective activities [179–181]. Additionally, mate has been developed as an herbal supplement to control body weight [182]. Long-term consumption of mate tea significantly increases serum insulin and ameliorates hyperglycemia and insulin resistance in mice [101]. Mate also induces significant decreases in food intake and weight gain in high fat diet-fed ddY mice. 3,5-*O*-dicaffeoyl quinic acid and matesaponin 2, two major constituents of mate, significantly elevated serum GLP-1 levels in ddY mice. However, neither inhibited DPP-4 activity [101]. Collectively, these findings suggest that mate and probably its active compounds act as an antidiabetic medicine through augmentation of GLP-1 production.

#### 4.5.7. Gingerol and Ginger

Ginger, *Zingiber officinale*, is commonly used as an ingredient in foods and medicine. Compelling data show that ginger extract has hypoglycemic, insulinotropic, and sensitizer effects on healthy humans and on experimental animals [103, 183–185]. More recently, Li and colleagues reported that ginger extract enhanced insulin release and reduced insulin resistance [103]. One clinical study reported that consumption of ginger powder, 3 g per day for 30 days, significantly reduced blood glucose and lipids in T2D patients [186]. Conversely, another study stated that consumption of ginger powder, 4 g daily for 3 months, did not alter blood sugar and lipids in patients with coronary artery disease [187]. This discrepancy may result from the variation in chemical composition of different ginger preparations. Gingerol and shogaol are the main active compounds in ginger extract. Gingerol was shown to attenuate sodium arsenite-induced T2D. This attenuation is related to islet-cell protection and increased insulin receptor signaling [102]. The role of shogaol in T2D treatment is not clear although this compound showed an elevation of glucose uptake in response to insulin in muscle and adipose cells [103]. 

#### 4.5.8. Epigallocatechin 3-Gallate (EGCG) and Chinese Tea

Chinese tea has been used as a beverage and food supplement since antiquity in China. It is made of the leaves and leaf buds of the *Camellia sinensis* species. One of the claimed health benefits of this tea is reduction of T2D risk and amelioration of T2D. Chinese green tea and oolong tea can prevent and/or ameliorate type 2 diabetes in humans [188–190] and experimental mouse models [191, 192]. EGCG, a major flavonol in tea, was shown to have antidiabetic activities in rodents [104, 105]. EGCG appears to have multiple antidiabetic actions including islet protection, increasing insulin secretion, decreasing insulin tolerance, and decreasing gluconeogenesis and insulin-mimetic action [104–106]. The role of EGCG in islet protection was shown to protect against *β*-cell death mediated by islet amyloid polypeptide (IAP) *in vitro* [193]. EGCG was reported to activate AMPK in adipocytes [95].

#### 4.5.9. *Ishige okamurae*

*I. okamurae*, an edible brown seaweed, lowers blood glucose in diabetic db/db mice [88]. Its mode of action involves reduction of insulin resistance and regulation of the hepatic glucose metabolic enzymes [88]. Diphlorethohydroxycarmalol, a phlorotannin of *I. okamurae*, inhibits the activity of *α*-glucosidase and *α*-amylase. This compound also decreases postprandial blood glucose level in streptozotocin-treated or normal mice [107]. 

#### 4.5.10. Soybean

Soybeans are thought to be an important protein source for food. Soybean isoflavones have been reported to treat atherosclerosis, cancer, osteoporosis, and others [194]. In addition, soy protein and isoflavonoids in soybeans improve insulin resistance and enhancement of insulin release [195, 196]. Genistein is a key isoflavone present in soybean (*Glycine max*) and other edible plants and has been reported to have anticancer, antioxidant, anti-inflammatory, and antiosteoporosis activities. More recently, genistein has been reported to treat obesity and diabetes [197]. This compound preserved islet mass by increasing *β*-cell count, proliferation, and survival in the pancreas [108, 109]. The data demonstrated that genistein could prevent T2D via a direct protective action on *β* cells without alteration of periphery insulin sensitivity [108]. Moreover, its antidiabetic mechanism involves activation of protein kinase A (PKA) and extracellular-signal-regulated kinases (ERK)1/2. However, another review stated that genistein could activate AMPK and, in turn, led to a reduction in insulin sensitivity [95, 110, 111]. Genistein improved diabetic complications such as vascular dysfunction and wound healing [198, 199]. In a clinical trial, genistein and/or soybean extract reduced the risk of T2D in overweight women [200]. 

Of note, soybean has been demonstrated to promote the secretion of insulin and GLP-1 [201]. Glyceollins, the phytoalexins produced by soybeans in response to fungi, were demonstrated to reduce hyperglycemia. These compounds could improve glucose-stimulated insulin secretion and prevent apoptosis and dysfunction in *β* cells in the presence of palmitate [112]. Accordingly, glyceollins enhanced GLP-1 secretion in NCI-H716 cells, an intestinal enteroendocrine L cell line [112]. Further, the antidiabetic action of glyceollin-rich soybean extract was confirmed in diabetic mice [121]. 

In conclusion, soybean and/or its active components can treat T2D via multiple pathways mainly involving insulin resistance, *β*-cell function, and GLP-1 production.

#### 4.5.11. Rooibos

Rooibos (*Aspalathus linearis*) is endemic to South Africa. It can be used as a herbal tea to treat diabetes in STZ-treated rats [114]. Aspalathin, a dihydrochalcone C-glucoside of rooibos, reduced hyperglycemia and ameliorated glucose intolerance through increased glucose uptake and insulin secretion in db/db mice [113]. Rutin, quercetin-3-*O*-rutinoside, is an inhibitor of *α*-glucosidase [115]. Rutin also decreased plasma glucose levels and increased insulin release in STZ-treated rats [116]. An aspalathin/rutin mixture at a ratio of 1 : 1 synergistically reduced blood glucose level in diabetic rats induced by STZ [114]. Additionally, rutin increased glucose uptake in hepatocytes *in vitro* and in mice *in vivo*, implying the function of rutin in insulin resistance [117]. Taken together, rooibos and probably its active compounds can treat T2D via targeting insulin tolerance, *β*-cell function, and inhibition of *α*-glucosidase.

#### 4.5.12. *Aloe vera*

 Extract of *A. vera* reduces hyperglycemia and hypercholesterolemia in diabetic patients [202, 203]. Similar antidiabetic effects were observed in alloxan- and STZ-treated animal models [204–206]. Aloeresin A, an active compound of *A. vera*, inhibited *α*-glucosidase activity [118]. *A. vera* and probably its active compounds exert their antidiabetic actions via inhibition of *α*-glucosidase and intestinal glucose absorption. In addition, extract of *A. vera* resulted in a reduction of hyperglycemia and insulin resistance [207]. As a whole, *A. vera* and its active components may treat diabetes via suppression of *α*-glucosidase activity (gut glucose absorption) and insulin resistance.

#### 4.5.13. Quercetin

Quercetin is a flavonoid that is widely distributed in plants and their products. It is commonly used to treat inflammation, viral infections, cancers, and metabolic syndrome. Early studies indicated that quercetin can treat T2D in STZ- and alloxan-treated mouse models and db/db mice [119, 120, 208, 209]. More recently, this compound was shown to lower fasting and postprandial blood glucose levels in diabetic db/db mice without any alteration in serum insulin level [210]. Moreover, like acarbose, quercetin inhibited *α*-glucosidase activity [211]. On the other hand, quercetin also suppressed DPP-4 activity *in vitro* [208]. However, it remains unclear whether this compound can stimulate GLP-1 production *in vivo*. To sum up, the data suggest that quercetin, a flavonoid commonly found in plants, controls glycemic control via reduction of intestinal glucose absorption and, probably, GLP-1 secretion.

#### 4.5.14. Resveratrol

Resveratrol is a stilbene compound, commonly found in plants and their products. It has a broad spectrum of bioactivities such as hepatoprotective, anticancer, anti-inflammatory, immunomodulatory, antidiabetic, and other activities [212]. Resveratrol has been demonstrated to treat diabetes [213, 214] and related complications [215–220] in different rodent models. When used as a T2D therapy evidence suggests that resveratrol exerts its action through multiple mechanisms. First, this compound can activate AMPK and the downstream molecules, leading to diminution of insulin resistance in db/db mice [121, 122]. It also prevented cell death of pancreatic *β* cells induced by IAP in culture [123] and in STZ-treated mice [221]. In addition, resveratrol enhanced glucose-mediated insulin secretion in *β* cells via the activation of SIRT1 [124], one of the cellular targets of resveratrol [222, 223]. A clinical study indicated that resveratrol can improve glycemic control in T2D patients [125]. Collectively, resveratrol ameliorates T2D and complications via the regulation of insulin resistance and *β*-cell functions.

#### 4.5.15. Coffee

Coffee is one of the most commonly consumed drinks worldwide. Recently, several studies have demonstrated an association between coffee intake and improvement in glucose tolerance and insulin sensitivity and a lower risk of T2D [224]. However, the active compound(s) and responsible target(s) are poorly understood. Accumulating data imply that constituents other than caffeine are active in glycemic control and/or insulin sensitivity. A study on people who consumed caffeinated and decaffeinated coffee showed no difference in the risk of T2D and insulin sensitivity in those drinking either type of coffee after 8 weeks of consumption [225]. However, caffeine improved the function of adipocytes and the liver [225]. Coffee is one of the major sources of dietary antioxidants. Roasting at high temperature can convert chlorogenic acid into quinides, which are known to reduce blood glucose levels in animal models [126]. In addition, coffee consumption might also mediate levels of GLP-1 [127]. Taken together, adequate coffee consumption is beneficial for T2D and its complications.

#### 4.5.16. Therapeutic Application

The paradigm of antidiabetic therapy has shifted from monotherapy to combination therapy. So far, no antidiabetic agents, used alone or in combination, have been able to cure this disease in humans. A major challenge in the search for efficacious therapies is that the molecular basis of T2D etiology has not yet been fully deciphered. Insulin resistance, *β*-cell function, glucose (re)absorption in the gut and kidney, and incretin production are the primary targets of current drugs. Compelling data on T2D treatment suggest that multiple targeting of the previous metabolic pathways is a plausible, albeit not yet fully developed approach to reversing T2D. Pharmacological interference of these targets with antidiabetic agents has undesirable side effects. Due to the richness and complexity of the compounds in plants, herbal therapy has always been thought to act on multiple targets in the human body. Even one single compound can have multiple targets, as shown by the role of quercetin in inhibition of DPP-4, *α*-glucosidase, and other enzymes. Multiple targeting is a double-edged sword in diabetes therapies. The multiple targets associated with antidiabetic herbal medicine make clinical trials complicated, but such an approach is more likely to lead to an eventual cure for T2D. In this review, the antidiabetic potential of the selected glucose-lowering herbs and their different mechanisms of action were summarized and discussed. The up-to-date information presented can be considered a cornerstone for further basic research and investigation into clinical applications of medicinal plants as T2D therapies.

## 5. Conclusions and Perspective

T2D, a disease known to man for many millennia, causes serious morbidity and mortality in humans. Despite significant progress in T2D and the development of antidiabetic drugs, no cures have been found. Medicinal herbs, long used in alternative and complementary medicine systems, are an extremely rich source of T2D remedies. Currently, understanding of the mechanisms through which herbal therapies mediate T2D is evolving, and they are generally being viewed as modulating of multiple metabolic pathways. Based on safety and their multiple targeting actions, herbal therapies are potent therapeutic means in T2D. Here, we summarized the chemistry and biology of nearly 40 extracts and compounds of plant origin that have been demonstrated to prevent and treat T2D via the regulation of insulin resistance, *β*-cell function, incretin pathways, and glucose (re)absorption. In addition, the actions, mechanisms and therapeutic potential of plant compounds and/or extracts, and new insights into the advantage of herbal therapy, which simultaneously governs distinct metabolic pathways immune cells and *β* cells, were discussed for T2D. Systematic information about the structure, activity, and modes of action of these plants and compounds will pave the way for research and development of antidiabetic drugs.

## Figures and Tables

**Table 1:** Active compounds and biological actions of antidiabetic herbs.

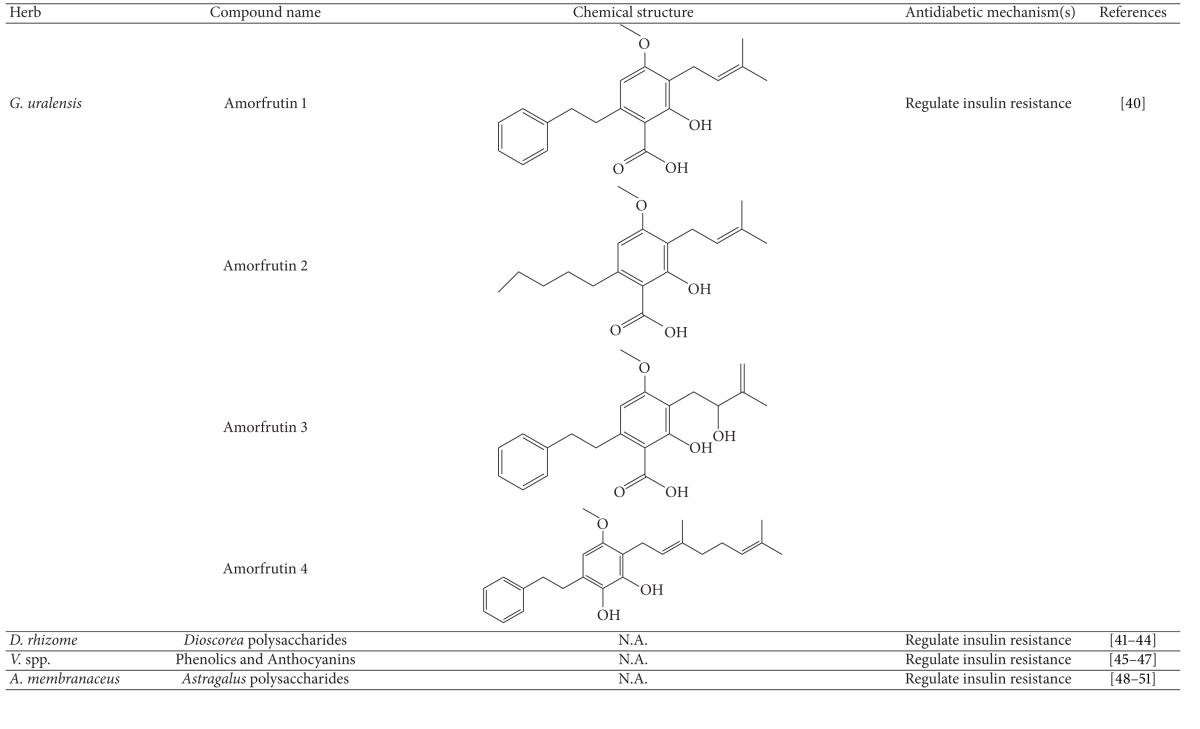 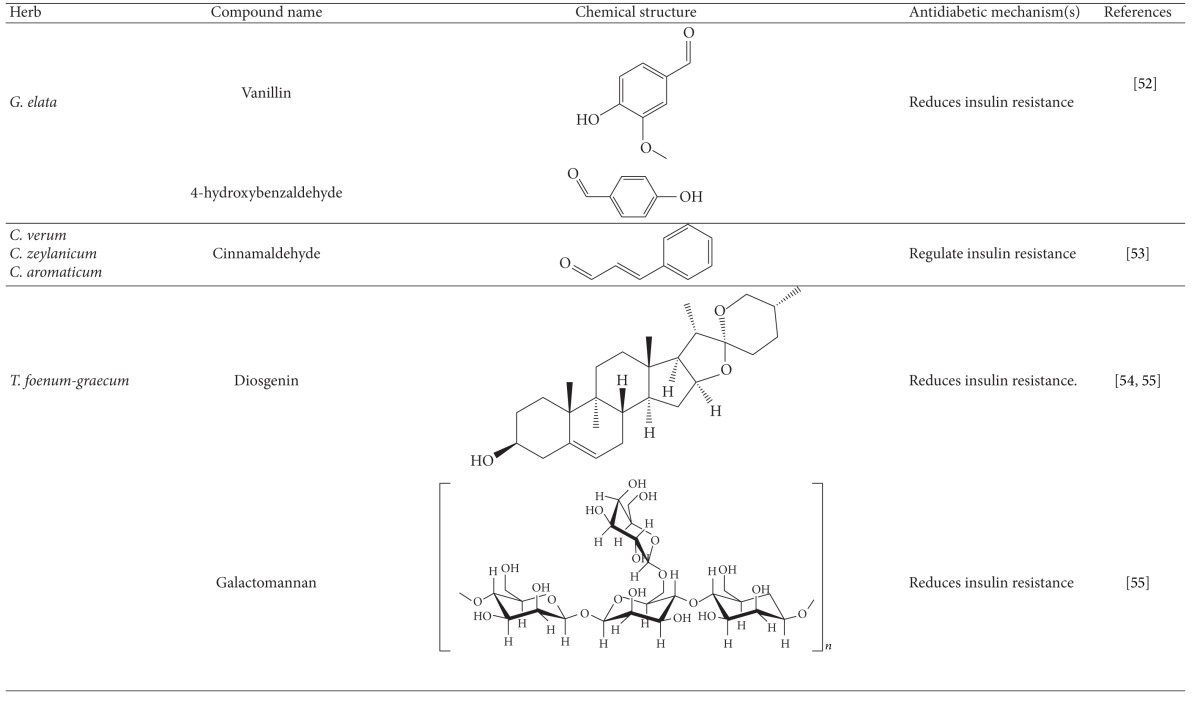 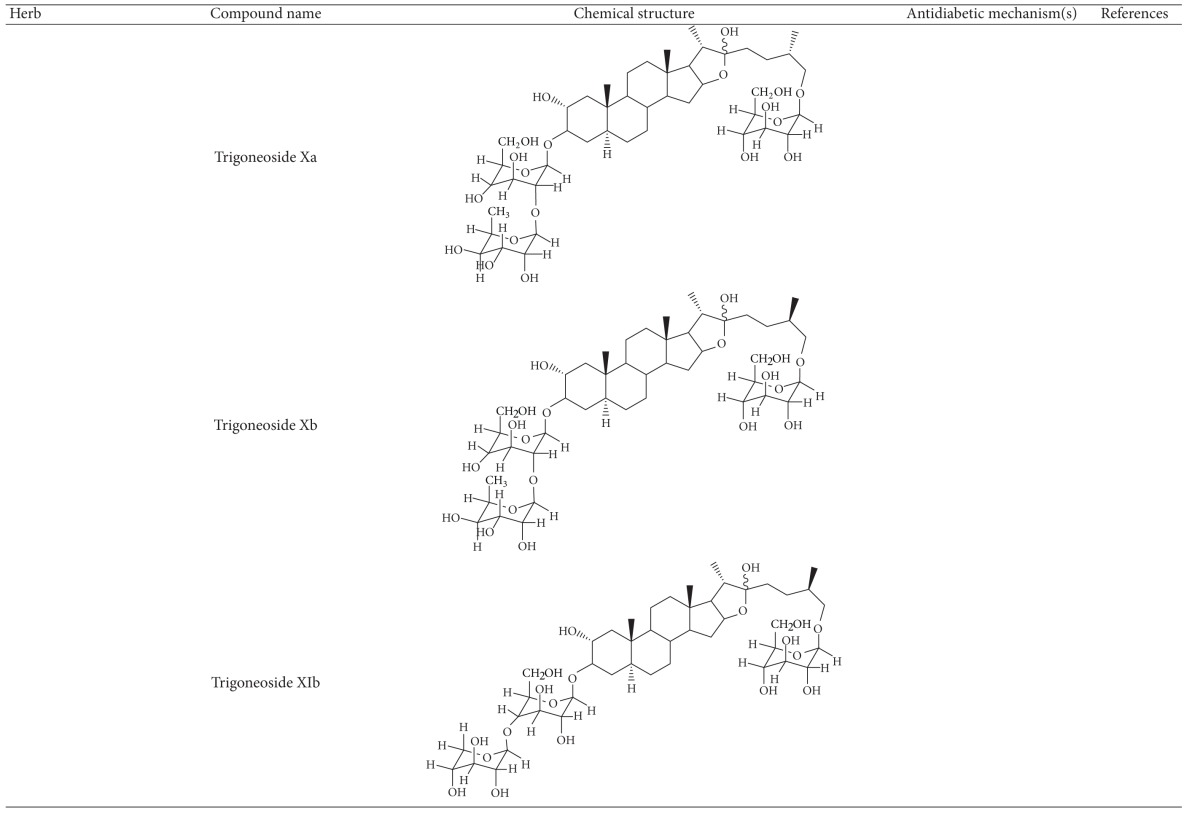 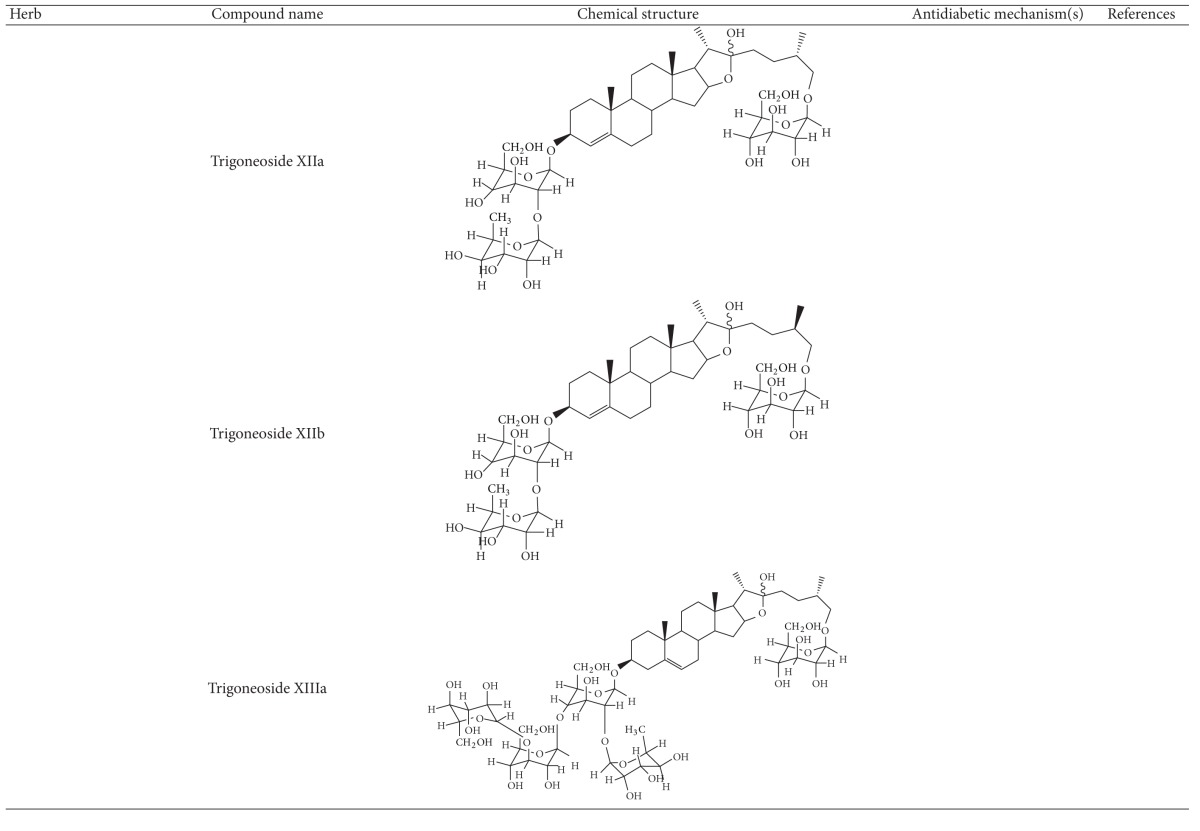 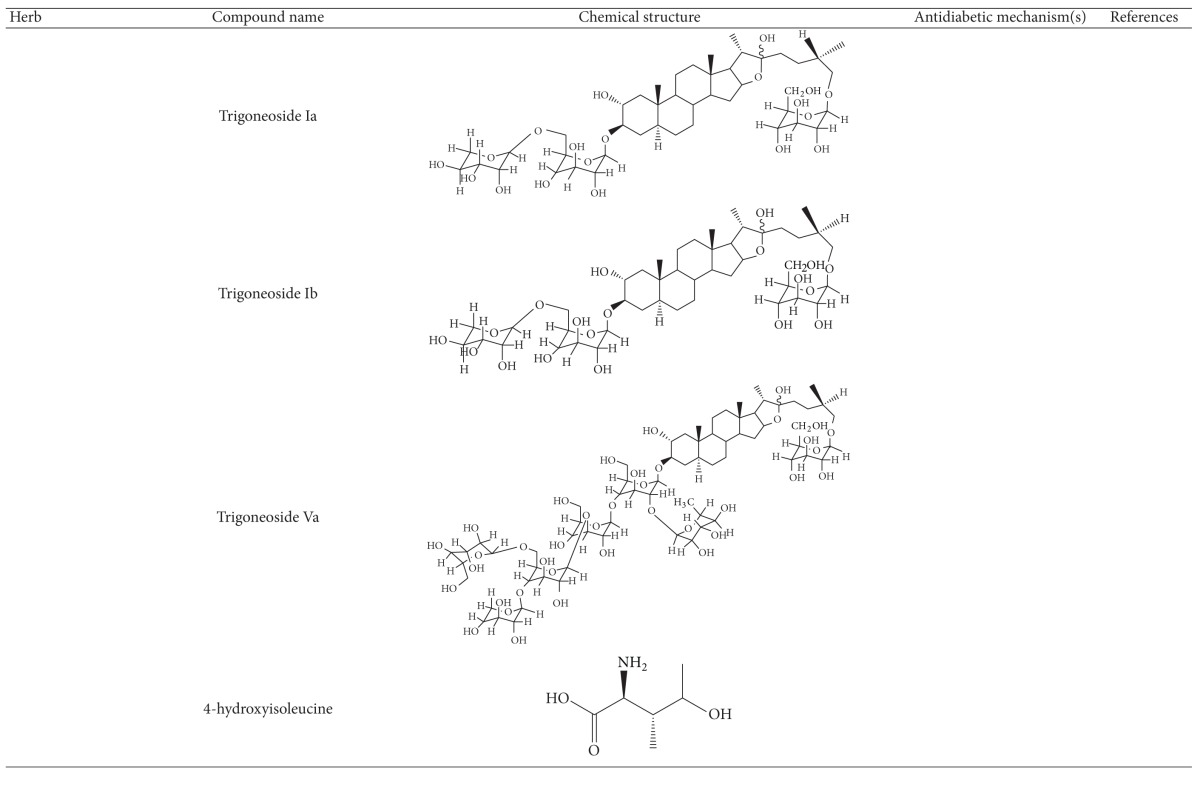 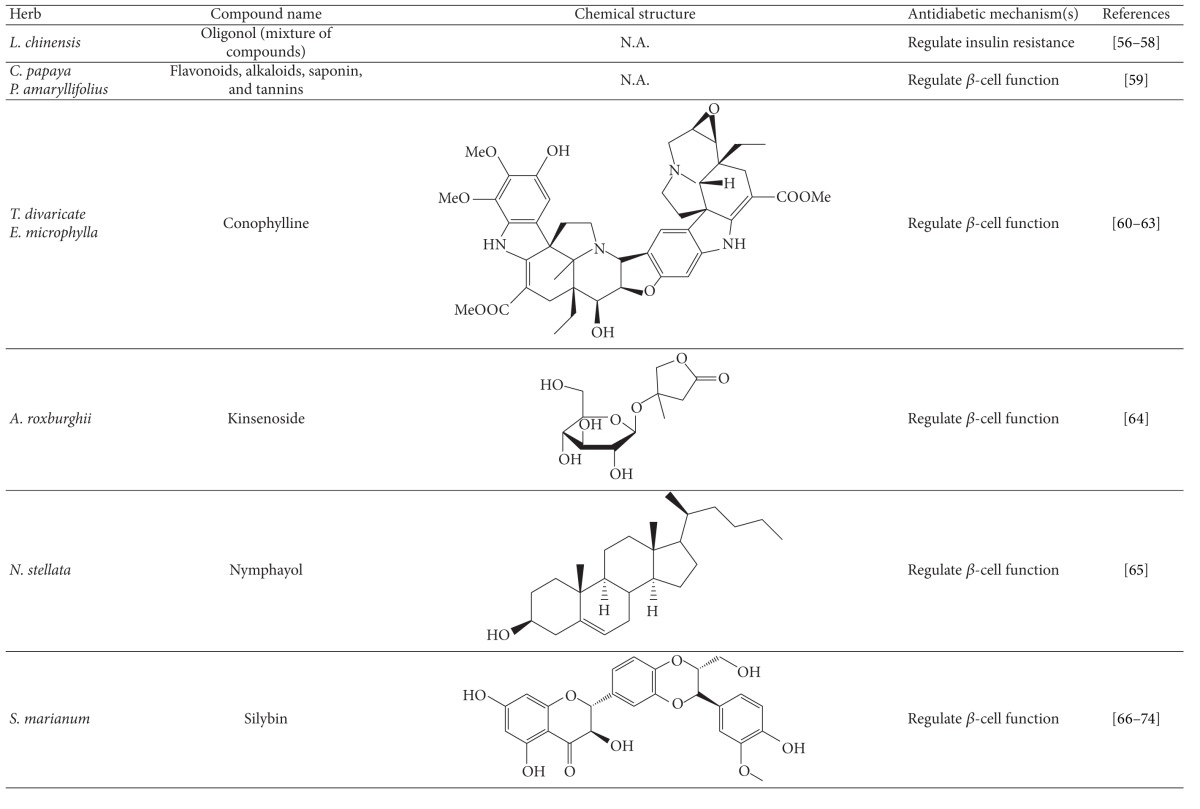 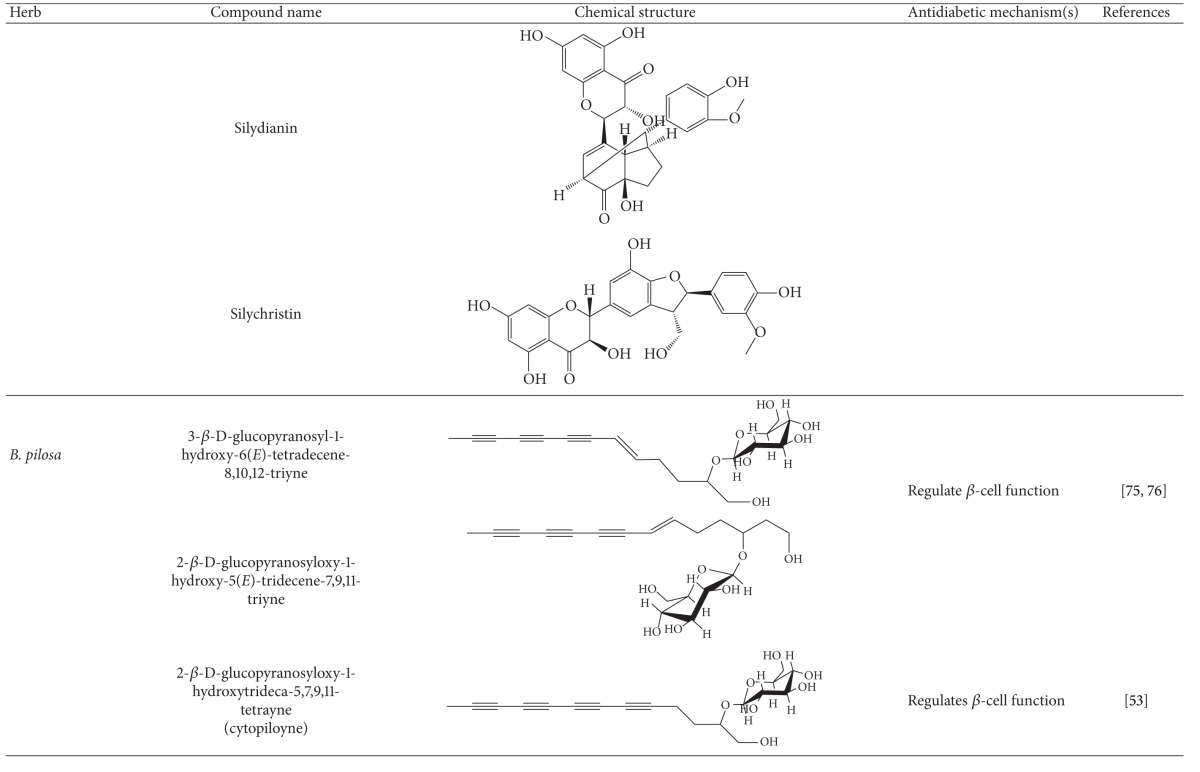 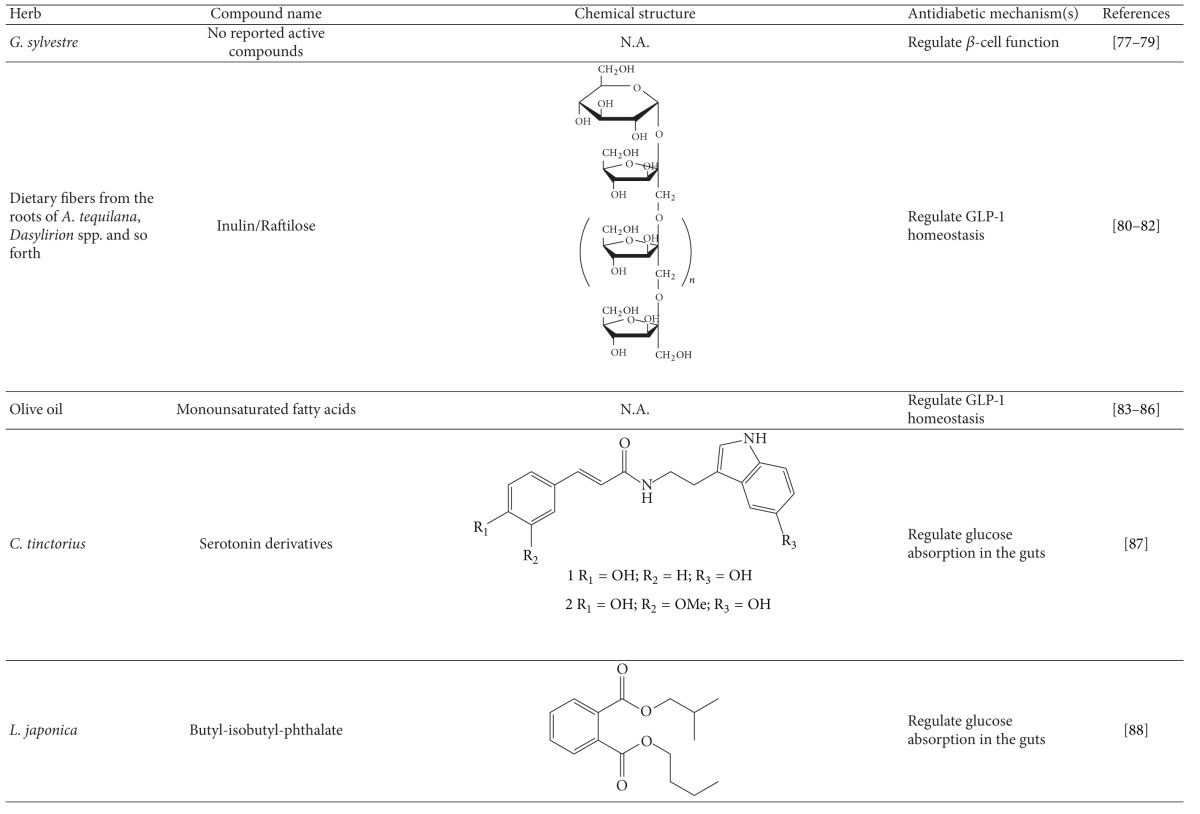 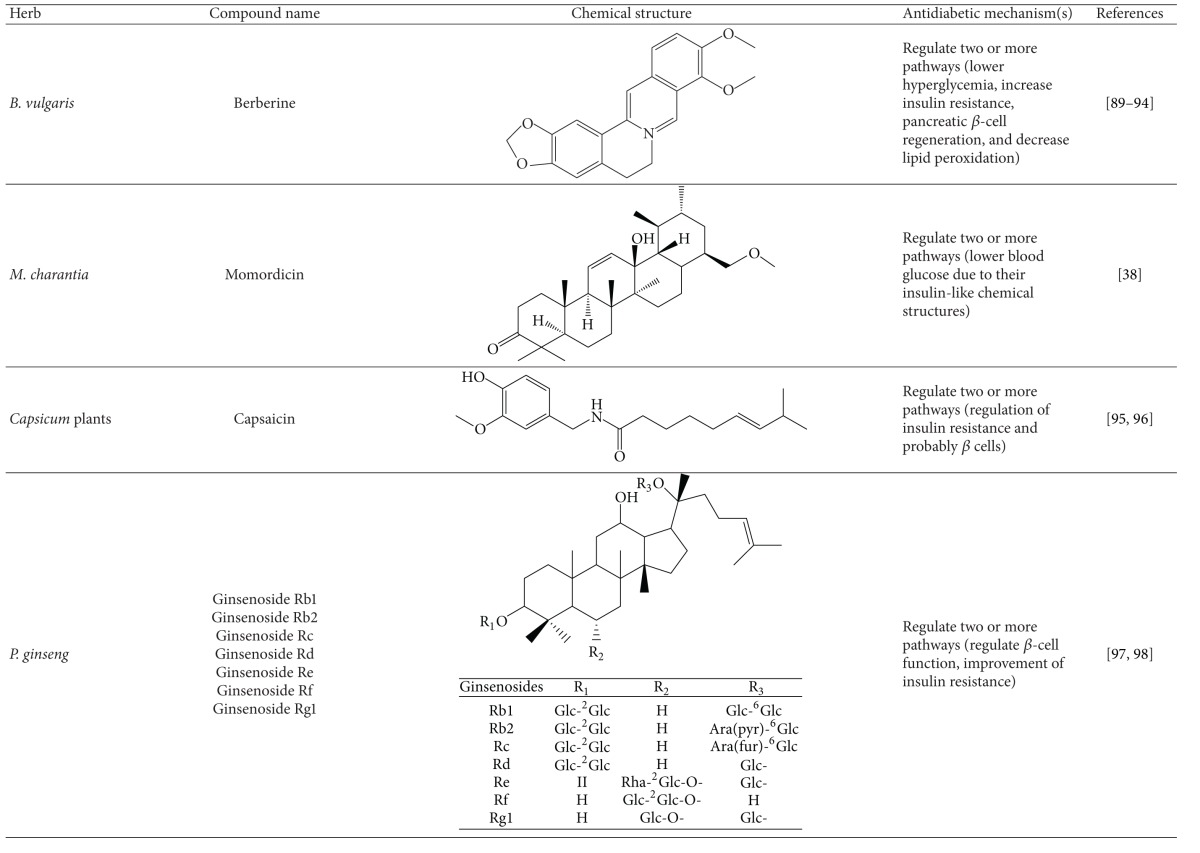 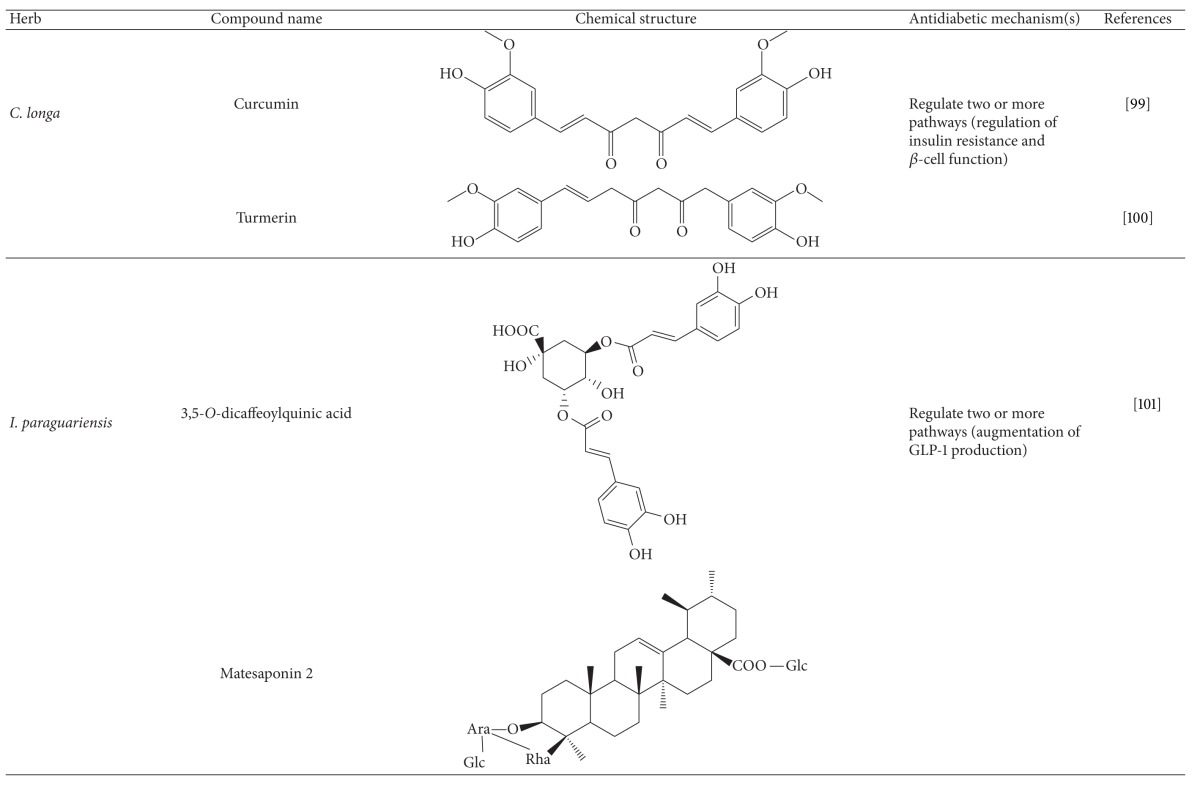 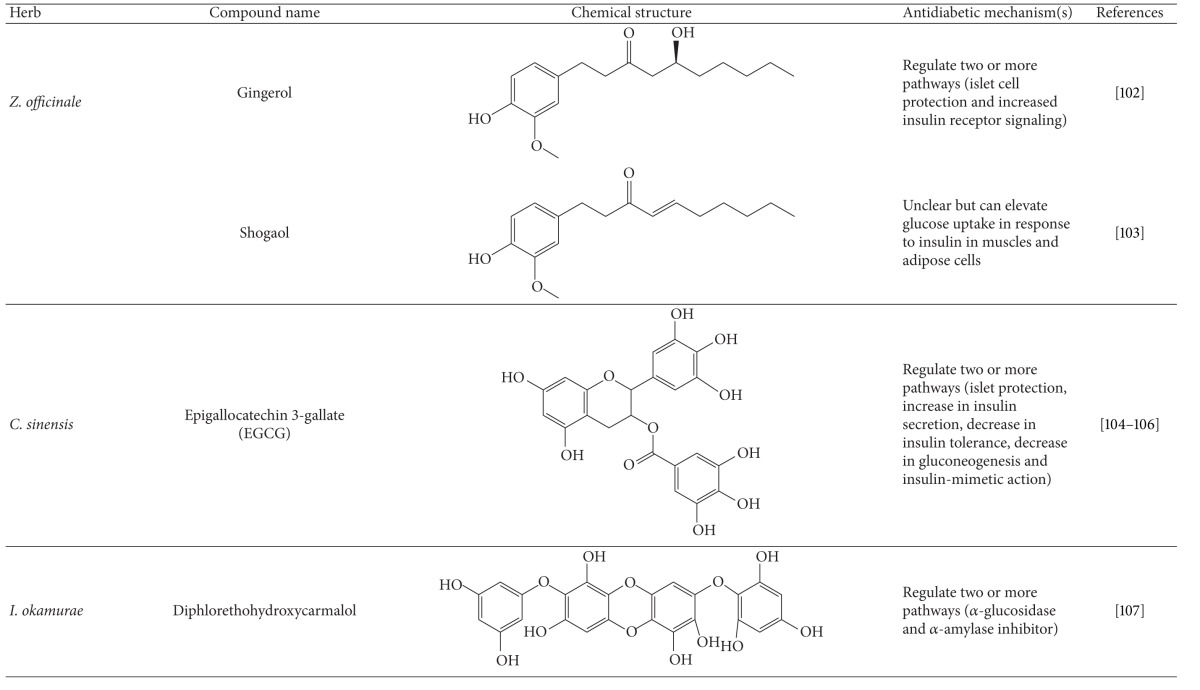 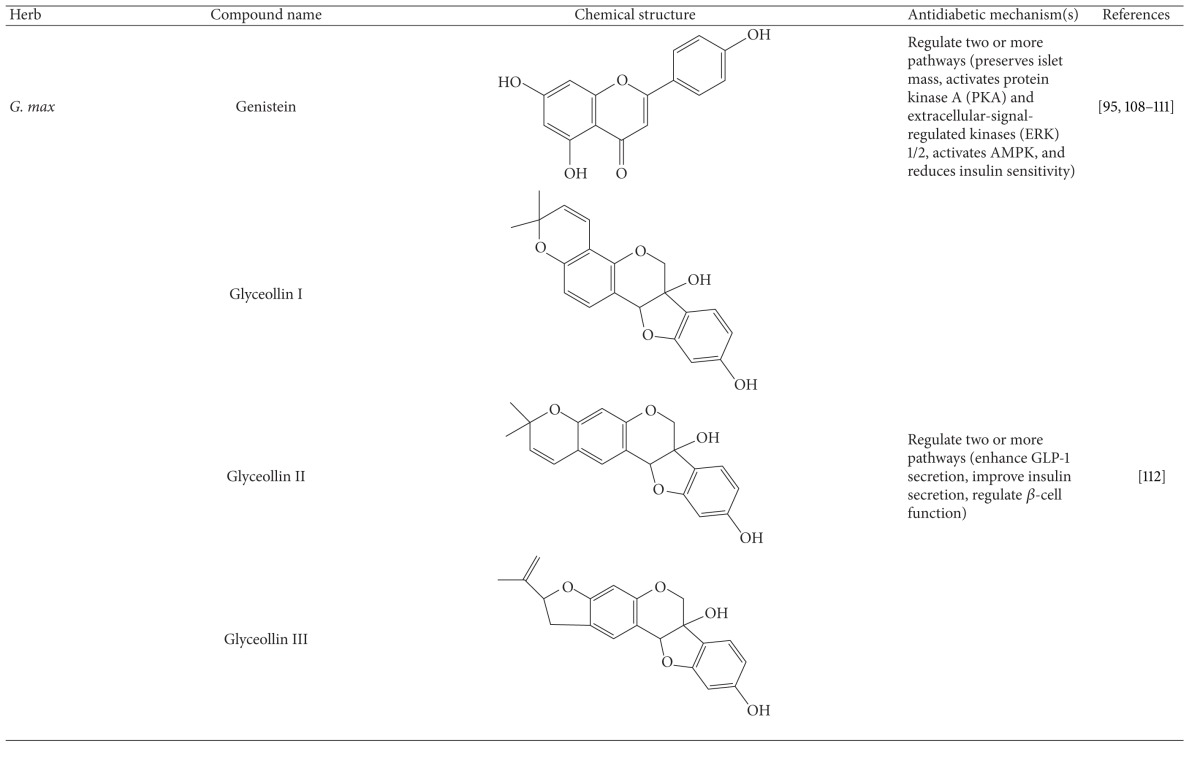 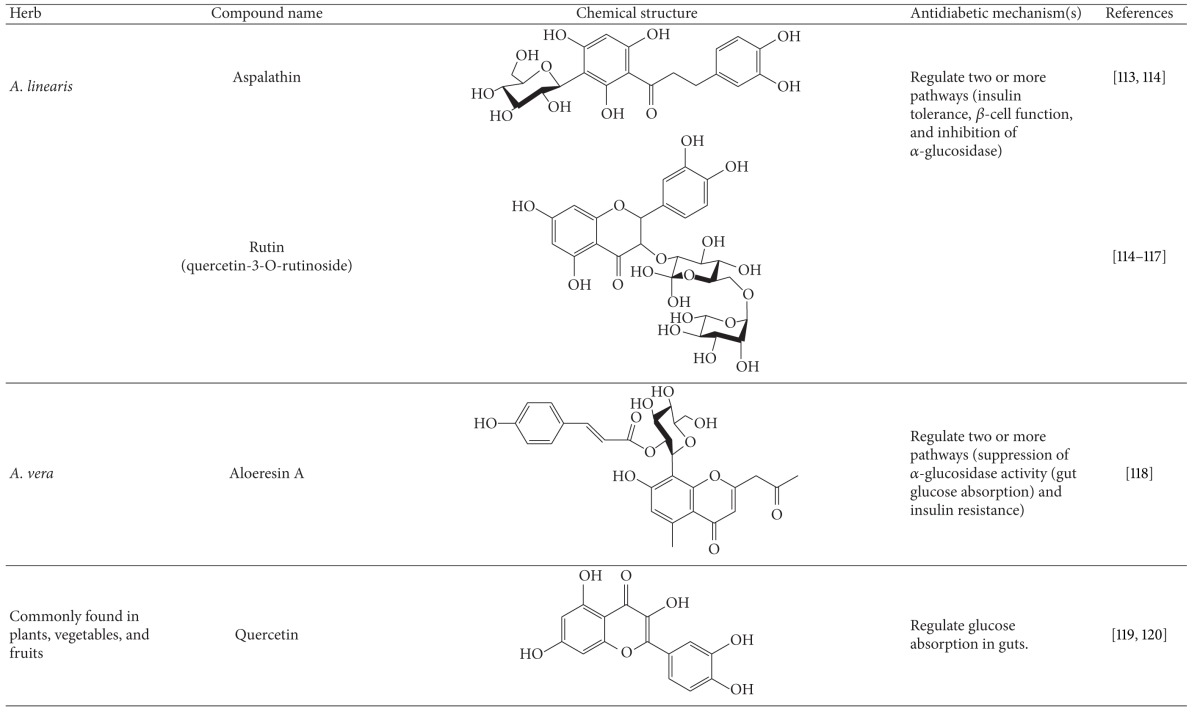 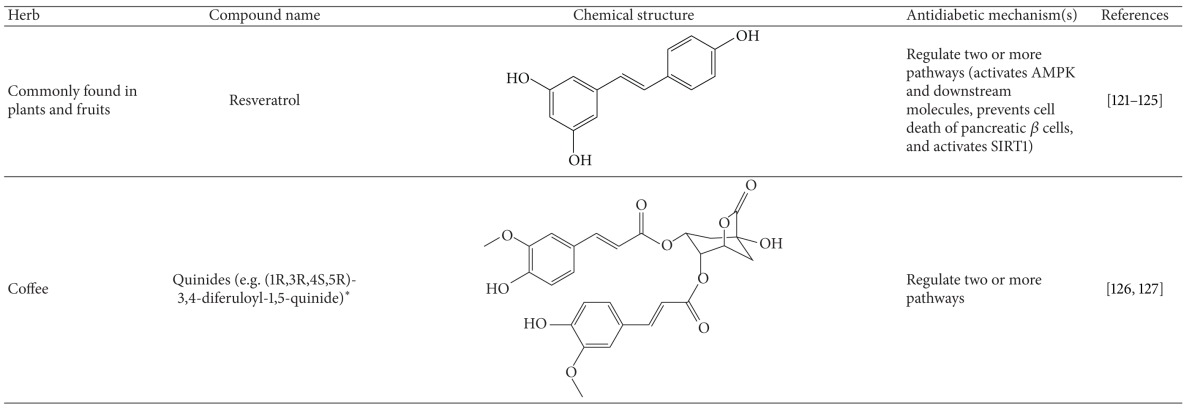

N.A.: not applicable.

*Quinides are derived from chlorogenic acid during roasting.
